# The Composition-Dependent Photoluminescence Properties of Non-Stoichiometric Zn_x_Ag_y_InS_1.5+x+0.5y_ Nanocrystals

**DOI:** 10.3390/mi10070439

**Published:** 2019-07-01

**Authors:** Jian Feng, Xiaosheng Yang, Rong Li, Xianjiong Yang, Guangwei Feng

**Affiliations:** 1 State Key Laboratory of Functions and Applications of Medicinal Plants, Guizhou Medical University, Guiyang 550014, Guizhou, China; 2 Department of Chemistry, School of Basic Medical Science, Guizhou Medical University, 9 Beijing Road, Guiyang 550004, Guizhou, China

**Keywords:** nanoparticles, luminescence, non-stoichiometric Zn_x_Ag_y_InS_1.5+x+0.5y_ nanocrystals, photoluminescence properties, tunable fluorescence emission, one-pot approach

## Abstract

A facile hot injection approach to synthesize high-quality non-stoichiometric Zn_x_Ag_y_InS_1.5+x+0.5y_ nanocrystals (NCs) in the size range of 2.8–3.1 nm was presented. The fluorescence spectra had single band gap features, and indicated the formation of alloy states rather than simple composite structures. The chemical compositions, photoluminescence (PL) emission wavelengths, and quantum yields of Zn_x_Ag_y_InS_1.5+x+0.5y_ nanocrystals were significantly influenced by the concentration of an organic capping agent. The appropriate proportion of 1-dodecanthiol in the precursor prevented the precipitation, increased the fluorescence quantum yield, and improved their optical properties. The proper ratio of capping agent allowed Zn, Ag, and In to form a better crystallinity and compositional homogeneity of Zn_x_Ag_y_InS_1.5+x+0.5y_ nanocrystals. The photoluminescence was tunable from blue to red in the range of 450–700 nm as the Ag content changed independently. The PL and absorption spectra of Zn_x_Ag_y_InS_1.5+x+0.5y_ nanocrystals showed a significant blue shift with the decrease of Ag content in the precursor. As there were no obvious differences on the average particle sizes of Zn_x_Ag_y_InS_1.5+x+0.5y_ samples, these results fully revealed the composition-dependent photoluminescence properties of Zn_x_Ag_y_InS_1.5+x+0.5y_ nanocrystals. The relative quantum yield reached 35%. The fluorescence lifetimes (τ_1_=115–148 ns and τ_2_=455–483 ns) were analogous to those of AgInS_2_ and (AgIn)_x_Zn_2(1−x)_S_2_.

## 1. Introduction

Ternary I-III-VI_2_ nanocrystals (NCs), including AgInS_2_ (AIS) and CuInS_2_ (CIS), have been developed recently to replace the toxic Cd- and Pb-contained NCs as fluorescence label and probes for biomedical and biological investigations [1,2,3,4]. These semiconductor nanomaterials have many advantages, such as high absorption coefficient, low toxicity, high energy conversion efficiency, and stability to solar radiation [5]. Therefore, they have great potential applications in photoelectric conversion and photocatalysis. These NCs have also been used in light-emitting diodes [6] and solar cells [7]. I-III-VI_2_-based ternary and quaternary semiconductors, such as CuInSe_2_ [8], CuGaSe_2_ [9], CuGa_x_In_2-x_S_3.5_ [10], and CuGa_1-x_In_x_Se_2_ [11], have been synthesized. In recent years, ternary AIS has become one of the research focuses in quantum dot synthesis and application investigation. The AIS nanoparticles possess a tunable band gap from 1.87 to 2.03 eV, which matches the solar spectrum very well and does not contain elemental Se. It can be used as a photoelectric conversion material to fabricate high-efficiency solar cells [12]. In addition, the fluorescence emission peak of AIS is also adjustable from visible to near infrared, and it possesses good photostability and large absorption coefficient, making it suitable for lighting and bio-imaging in vivo and in vitro [13,14].

AIS nanocrystals have been synthesized by several methods, such as thermal decomposition of sulfur-containing complexes of different metals or direct reaction of sulfur with metal ions in the presence of coating agents [15]. In the synthetic reaction, metal ions usually form metal complexprecursors with amines or dodecyl mercaptan to ensure controlled growth during the synthesis process. Sulfur powder, mercaptan, dithiocarbamate, or carbon disulfide was used as sulfur sources. Metal mercaptan and dithiocarbamate were the single precursors to provide both metal ions and sulfur elements. The optical properties of bulk AgInS_2_ were studied in 1976 [16]. Redjai et al. [17] investigated the donor–acceptor pair transition in bulk AgInS_2_ by time-resolved spectroscopy, and obtained the thermal quenching curve of its emission. The effect of defects in bulk and AgInS_2_ film, such as vacancies and interstitial atoms, has also been carefully tested [18,19,20]. Recently, it has been observed that AgInS_2_ nanocrystals have wide fluorescence emission peaks and relatively large Stokes shifts. It has been suggested that its fluorescence emission was caused by carrier recombination captured by energy levels in the band gap formed by structural defects. It was the characteristic of donor–acceptor pair transition [21].

However, the AIS core has relatively lower photoluminescence (PL) quantum yields (QYs) [22]. Over the last decade, the quantum yields of AIS have been enhanced by two approaches. The first method is the synthesis of core–shell structure coated with a ZnS shell [23,24,25]. More reports demonstrated the improvement of QYs for core–shell AIS NCs with smaller cores synthesized at lower reaction temperatures. However, smaller cores decreased the crystallinity and increased surface defects, leading to a cumbersome experimental procedure [26]. The second strategy for increasing QYs and tuning photoluminescence (PL) of AIS NCs involved alloyed AIS with ZnS to form Zn-Ag-In-S solid solutions at a higher temperature [26,27,28]. A series of (AgIn)_x_Zn_2(1-x)_S_2_ solid solution materials with different compositions have also been reported. Unlike the AgInS_2_/ZnS core–shell structure, in solid solution nanocrystals, Zn was doped into the crystal structure and participated in the formation of conduction bands. The density functional theory (DFT) results showed that the 4d orbital of Ag and the 3p orbital of S formed valence bands of (AgIn)_x_Zn_2(1-x)_S_2_ [29]. The 5s5p orbital of In and 4s4p orbital of Zn participated in the formation of conduction bands. The highest occupied molecular orbital (HOMO) and the lowest unoccupied molecular orbital (LUMO) orbits of solid solution crystals are located between ZnS and AgInS_2_. By changing the ratio of AgInS_2_ to ZnS in solid solution, the valence band and conduction band can be changed continuously. Increasing the amount of Zn in AIS directly resulted in a large blue shift of the PL emissions [30], which is ascribed to the increase of the band gap caused by the zinc being incorporated into the AIS core. The alloyed layer abated the lattice mismatch between AIS and ZnS, reduced surface defects, and improved PL properties. Therefore, the band gap and PL emission of AIS-based NCs can be conveniently adjusted by changing their composition and size.

So far, a large number of reports have focused on the preparation and the application of Zn-Ag-In-S NCs. Most of the synthetic methods concentrated on the thermolysis of (AgIn)_x_Zn_2(1-x)_(S_2_CN(C_2_H_5_)_2_)_4_ precursors to form (AgIn)_x_Zn_2(1−x)_S_2_ NCs [31,32,33,34]. In these reports, the molar ratio of (Zn + Ag) to In was varied to achieve the different PL emissions, in which the relative dose of Zn and Ag remained constant. In our previous work [35], high-quality non-stoichiometric Zn_x_Cu_y_InS_1.5+x+0.5y_ NCs were synthesized using a one-pot strategy, which exhibited excellent PL QYs and tunable PL in 450–640 nm by holding Zn (mole% = 20–35%), In, and S constant and changing the mole percentage of Cu in the precursor. For the prepared (ZnCu)_x_InS_2_ nanoparticles in the same reaction condition, the PL emissions were in the range of 560–640 nm and the PL intensities were lower than those of the Zn constant (25%) samples. Here, we extend this one-pot strategy to fabricate the non-stoichiometric Zn_x_Ag_y_InS_1.5+x+0.5y_ nanocrystals (ZAIS NCs). This is the first exploration of the synthesis of ZAIS NCs with Zn constant (25%) and a different molar ratio of Ag to In, which exhibits composition-dependent photoluminescence properties with high PL QYs and tunable PL wavelength.

## 2. Materials and Methods 

In(Ac)_3_ (99.99%), sulfur powder (S, 99.99%), Zn(Ac)_2_ (99.99%), Ag(Ac) (99.99%), oleic acid (OA, 90%), octadecene (ODE, 90%), and 1-dodecanthiol (DDT, 99.9%) were purchased from Sigma-Aldrich (St. Louis, MO, USA). Nitric acid (70%), methanol (AR), and chloroform (AR) were purchased from Beijing Chemical Reagent Ltd., Beijing, China. All chemicals were used without further purification.

Zn_x_Ag_y_InS_1.5+x+0.5y_ (ZAIS) NCs were prepared using our previous methods [35] with a slight modification. For a typical synthetic reaction, 0.01 mmol Ag(Ac), 0.025 mmol Zn(Ac)_2_, 0.1 mmol In(Ac)_3_, 1 mmol DDT, 0.4 mmol OA, and 4 g of ODE were loaded in a round bottom flask. The mixture was heated at 180 °C for 30 min to form a clear solution and then kept at 260 °C for 10 min. A sulfur solution of ODE (0.3 mmol)was injected into the three-neck flask and heated for 10 min at 260 °C, followed by cooling to room temperature. The precipitation was obtained by centrifugation at 13,000 rpm for 15 min after adding 8-fold volume of methanol to the reaction solution and then the precipitation was redispersed in chloroform. The purification approach ofthe ZAIS NCs was carried out by repeating the above process several times. Changing the Ag dosage in the precursor, a series of ZAIS NCs with different Ag content (including 0.015, 0.01, 0.005, 0.0025, 0.00125, 0.000625, and 0 mmol, and denoted ZAIS-1 to ZAIS-7, respectively) was obtained. The chemical compositions of ZAIS-1 to ZAIS-7 were carried by inductively coupled plasma optical emission spectrometry/mass spectrometry (ICP-OES/MS, Thermo Jarrell-Ash Corporation, Franklin, MA, USA). The purified ZAIS NCs were first digested by nitric acid. The concentrations of In^3+^, Ag^+^, and Zn^2+^ ions in the clear digestion solutions were determined directly by ICP-OES/MS after being diluted with distilled water. The relative PL quantum yield of the ZAIS NCs was determined by comparing the integrated emission of the samples to that of Rhodamine 6G (Rhodamine 6G in ethanol, QY = 95%) solutions with the same optical density at the excitation wavelength and similar fluorescence wavelength.

Transmission electron microscope (TEM, Hillsboro, OR, USA) and high-resolution transmission electron microscope (HRTEM, Hillsboro, OR, USA) images were examined on an FEI Tecnai G2 F20 using 200 kV acceleration voltage. ZAIS NCs were dispersed in chloroform and then dropped on carbon-coated copper grids. X-ray powder diffraction (XRD) patterns were obtained from a Rigaku D/max-2500 X-ray diffractometer (Rigaku Corporation, Tokyo, Japan) with Cu Kα radiation (λ = 1.5418 Å). UV-vis absorption spectra were performed on a Cary 50 spectrophotometer (Varian, Salt Lake City, UT, USA). Fluorescence spectra were recorded on a LS55 luminescence spectrometer (PerkinElmer, Waltham, MA, USA). A Lecroy Wave Runner 6100 digital oscilloscope (Teledyne LeCroy, Chestnut Ridge, NY, USA) was employed to obtain the fluorescence decay curve. A Continuum Sunlite optical parametric oscillator (Teledyne LeCroy, Chestnut Ridge, NY, USA) was the excitation source with tunable laser (pulse width = 4 ns, gate = 50 ns).

## 3. Results and Discussion

**Figure 1:**
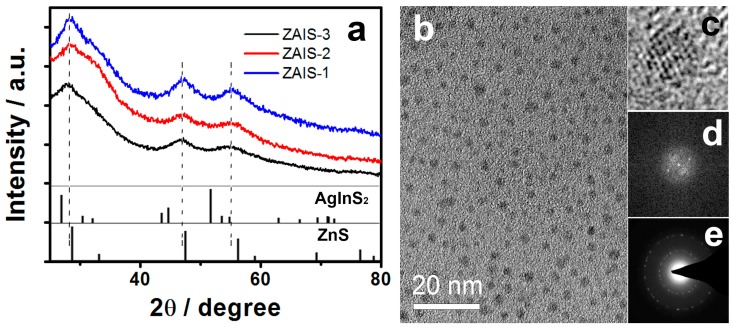
(**a**) X-ray powder diffraction (XRD) patterns of Zn_x_Ag_y_InS_1.5+x+0.5y_ nanocrystals (ZAIS NCs) (ZAIS-1, ZAIS-2, and ZAIS-3), (**b**) transmission electron microscope (TEM), (**c**) high-resolution transmission electron microscope (HRTEM) micrograph, (**d**) corresponding inverse fast fourier transform (FFT) pattern, and (**e**) energy dispersive X-ray spectrometry (EDX) analysis of ZAIS-2. The XRD patterns of AgInS_2_ (JCPDS 25-1330) and ZnS (JCPDS 65-1691) are shown as reference.

The XRD patterns of ZAIS-1, ZAIS-2, and ZAIS-3 areshown in Figure 1a. The broad peaks of the samples reveal the small-size nature of the NCs. Three diffraction peaks at 28.3, 47.2, and 55.2° areobserved between the peaks of AgInS_2_ and ZnS, and are practically located at the same diffraction angles of bulk ZnS. These diffraction peaks were attributed to the (111), (220), and (311) planes [28], respectively. This indicated that the XRD diffraction peaks did not originate from the mixture of AgInS_2_ and ZnS. These results demonstrated that the ZAIS NCs were not a simple mixture of AgInS_2_ and ZnS but the compositional homogeneous Zn_x_Ag_y_InS_1.5+x+0.5y_ solid solution [35]. Moreover, ZAIS-1, ZAIS-2, and ZAIS-3 presented similar diffraction patterns even though the Ag content was different in the precursor. These results indicated that the ZAIS NCs had monocrystalline structures which coincided with the HRTEM measurements (Figure 1c). One of the high-resolution TEM images of ZAIS-2 is shown in Figure 1c. It presents the continuous lattice fringes, which indicate the high crystallinity of the ZAIS-2 NCs. The TEM photographs (Figure 1b) of ZAIS-2 show that the nanoparticles are approximately spherical in shape and comparatively monodisperse. Average particle sizes of the ZAIS samples were 2.8–3.1 nm. The chemical compositions of the ZAIS NCs were determined by ICP-MS analysis. The analysis results areshown in Table 1. They indicate that the compositions of purified ZAIS NCs can be precisely controlled, which conforms particularly well to the cationic concentration in the precursor.

**Figure 2:**
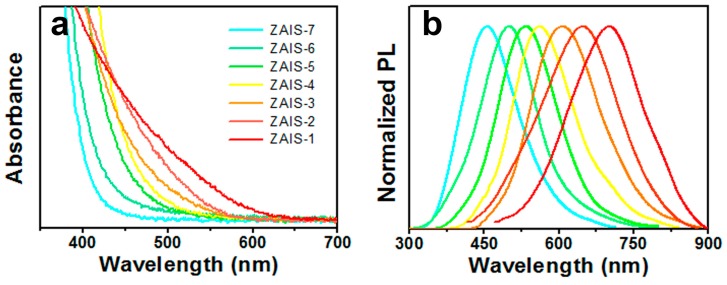
(**a**) UV-vis absorption spectra and (**b**) PL spectra of the ZAIS NCs (ZAIS-1 to ZAIS-7) in chloroform.

The absorption onsets of ZAIS (Figure 2a) were blue shifted from ZAIS-1 to ZAIS-7, indicating thatthe band gap energy (E_g_) of the ZAIS NCs was augmented with the decreasing Ag content. In the serial ZAIS NCs, while keeping In, S, and Zn (mole% = 20–35%) constant and changing the mole ratios of Ag in the precursor, the PL emission wavelengths were tunable from 450 to 700 nm. The PL wavelengths and corresponding chemical compositions of the ZAIS samples are shown in Table 1. The PL and absorption spectra of the ZAIS NCs showed a significant blue shift with the decrease of Ag content. As there were no obvious differences inthe average particle sizes of the ZAIS samples, these results fully revealed the composition-dependent photoluminescence properties of the ZAIS NCs. The onset wavelength varied from 620 to 450 nm. These results agreed well with the phenomena reported in our previous work [30]. The PL peaks of the ZAIS NCs exhibited full width at half maximum around 150 nm. They were remarkably broadened as compared to CdSe but identical to AIS-based NCs. The size-selective precipitation approach [22] was adopted to reduce the particle size distribution of the ZAIS NCs. However, the broadened features of the PL peaks were totally maintained, similar to CuInS_2_ NCs. This appeared to result from the donor–acceptor pair radiative recombination of charge carriers generated by surface defects and vacancies. The relative PL QYs of the ZAIS NCs are also shown in Table 1. The highest QY reached 35% for ZAIS-4. Most QYs of the ZAIS NCs were higher than those of AIS NCs reported in the literature.

I-III-VI_2_-based ternary and quaternary semiconductor NCs, including AgInS_2_, CuInS_2_, and Cu(In,Ga)Se_2_ reported previously, had no sharp exciton band. They all exhibited a broad and large absorption band in the visible to near-IR wavelength region. The displayed broad absorption spectra were also afeature of the ZAIS NCs, regardless of their particle sizes and chemical compositions [23]. The broad and large absorption band of the ZAIS NCs signified a high light absorption coefficient in the solar spectrum and highly efficient light energy conversion. Meanwhile, the large full width at half maximum resulted from the donor–acceptor pair radiative recombination of charge carriers generated by surface defects and vacancies. The precise adjustment of the ZAIS NCs could be advantageous to the synthetic control of surface defects, vacancies, and electronic energy structure and the further control of exciton behavior [36]. By precisely controlling the chemical composition of the ZAIS NCs, the effective transfer of photogenerated electrons and holes was induced, and the PL efficiency of the ZAIS NCs was improved.

The chemical compositions, PL emission wavelength, and QY of ZAIS-4 NCs were significantly influenced by the concentration of an organic capping agent in the precursor. The effects of different DDT dosages on the chemical composition and PL emission properties of the solid solution were investigated and are displayed in Table 2. DDT has been documented as an effective capping agent for adjusting the reactivity of metal ions [22]. The chemical composition of the purified ZAIS-4 NCs was decided by the dosage of DDT. The Ag content in the ZAIS-4 NCs was increased with the increase of the DDT dosage in the precursor (shown in Table 2). When DDT, in amounts greater or less than 1.0 mmol, was added to the reaction solution, a white precipitate was produced. This suggested that an appropriate DDT concentration was conducive to the formation of a better crystallinity and compositional homogeneity of the ZAIS NCs. The PL emission wavelength of the ZAIS-4 NCs was a blue shift with the decrease of DDT dosage in the precursor, which maybe related to the decrease of Ag content in the ZAIS-4 NCs. As shown in Table 2, the QY of the ZAIS-4 NCs was also significantly influenced by the dosage of DDT, which was the key factor in the synthesis of the ZAIS solid solution. The appropriate proportion of DDT in the precursor could prevent the precipitation in the reaction system, increase the fluorescence quantum yield, and improve their optical properties.

**Figure 3:**
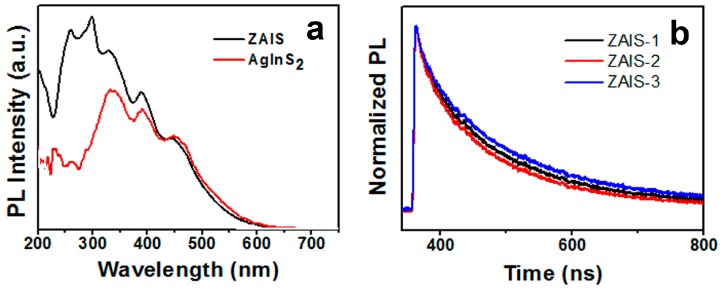
(**a**) PL excitation spectra and (**b**) PL decay curves of ZAIS-1, ZAIS-2, and ZAIS-3.

The excitation spectra of AgInS_2_ and the ZAIS NCs are shown in Figure 3a. Three excitation peaks of ZAIS at about 329 nm, 391 nm, and 459 nm were observed. This coincides with the three peaks of AgInS_2_, and a new excitation peak appeared between 226 and 319 nm. It was shown that Zn and Ag in the crystals recombined with the conduction band and valence band of the NCs, forming a new structure of the conduction band and valence band energy of the ZAIS NCs. This was similar to that of Zn-Cu-In-S crystals. Meanwhile, the fluorescence spectra of the ZAIS NCs had single band gap characteristics, and these evidences indicated the formation of alloy states rather than simple composite structures of In-S, Ag-S, and Ag-In-S.

Figure 3b shows the PL decay curves for the ZAIS-1, ZAIS-2, and ZAIS-3 NCs. These curves were well fitted by a biexponential function as the form I(t) = aexp(−t/τ_1_) + bexp(−t/τ_2_). Based on the curve-fitting method, the lifetimes of 115–148 ns (τ_1_) and 455–483 ns (τ_2_) were determined (see Table 3). The average lifetimes calculated according to the formula in the literature [37] were 194, 234, and 237 ns for Ag content of 0.015, 0.01, and 0.005 mmol in the precursors. The fluorescence lifetimes (τ_1_ and τ_2_) were analogous to those of AIS and (AgIn)_x_Zn_2(1−x)_S_2_ [38]. The components of 115–148 ns can be assigned to donor–acceptor pair recombination related to surface defects and vacancies. The components of 455–483 ns can be allocated to transitions of intrinsic donor–acceptor pairrecombination [34].

## 4. Conclusions

A facile one-pot hot injection approach to successfully synthesize high-quality non-stoichiometric ZAIS NCs in the size range of 2.8–3.1 nm was presented. XRD, HRTEM, and fluorescence spectra showed that Zn, Ag, In, and S formed the alloyed non-stoichiometric solid solution. The fluorescence spectra had single band gap features, and indicated the formation of alloy states rather than simple composite structures. The effects of different reaction conditions on the composition and PL emission properties of the solid solution were investigated. It was found that the appropriate ratio of DDT was beneficial to the formation of alloy state ZAISNCs, which was the key factor in the synthesis of the ZAIS solid solution. The appropriate proportion of DDT can prevent the precipitation in the reaction system, increase the fluorescence quantum yield, reduce their half-peak width, and improve their luminescence properties. The photoluminescence wastunable from blue to red in the range of 450–700 nm since the Zn and Ag contents in the precursor changed. The PL and absorption spectra of the ZAIS NCs showed a significant blue shift with the decrease of Ag content in the precursor. As there were no obvious differences in the average particle sizes of the ZAIS NCs amples, these results fully revealed the composition-dependent photoluminescence properties of the ZAIS NCs. The relative quantum yield reached 35%. The fluorescence lifetimes (τ_1_ = 115–148 ns and τ_2_ = 455–483 ns) were analogous to those of AIS and (AgIn)_x_Zn_2(1−x)_S_2_. The components of 115–148 ns can be assigned to donor–acceptor pairrecombination related to surface defects and vacancies. The components of 455–483 ns can be allocated to transitions of intrinsic donor–acceptor pair recombination.

## Figures and Tables

**Table 1:** Chemical compositions of the ZAIS NCs (ZAIS-1 to ZAIS-7) determined by inductively coupled plasma mass spectrometry (ICP-MS) analysis (normalized according to indium ion concentrations).

Sample	PL Emission Wavelength (nm)	Relative Quantum Yield	Silver Dosage in the Precursor y (%)	Composition of the Nanoparticle	
		(%)		Ag (%)	Zn (%)
ZAIS-1	700.5	8	15.0	14.1	22.9
ZAIS-2	647.5	15	10.0	9.12	23.1
ZAIS-3	607.0	30	5.00	4.47	24.9
ZAIS-4	561.0	35	2.50	2.33	24.1
ZAIS-5	533.5	23	1.25	1.15	23.5
ZAIS-6	500.5	11	0.625	0.564	24.1
ZAIS-7	456.0	5	-	-	24.4

**Table 2:** Chemical compositions and photoluminescence (PL) emission properties of ZAIS-4 NCs with different 1-dodecanthiol (DDT) dosage in the precursor (normalized according to indium ion concentrations).

Sample	PL Emission Wavelength (nm)	Relative Quantum Yield	DDT Dosage in the Precursor (mmol)	Composition of the Nanoparticle	
		(%)		Ag (%)	Zn (%)
Sample1	542.5	4	0.25	1.57	21.5
Sample2	550.0	12	0.5	2.04	23.7
Sample3	561.0	35	1.0	2.33	24.1
Sample4	572.0	18	1.5	2.55	26.2
Sample5	573.5	7	2.0	2.47	25.6

**Table 3:** Decay times and amplitude constant ratios of ZAIS-1, ZAIS-2, and ZAIS-3.

Sample	ZAIS-1	ZAIS-2	ZAIS-3	(AgIn)_x_Zn_2(1−x)_S_2_
τ_1_/ns	115	146	148	127–131
a	0.423	0.562	0.577	-
τ_2_/ns	455	481	483	538–655
b	0.0324	0.0615	0.0641	-
average	194	234	237	-
